# Supplementary material for: Spatial mapping of immune cell environments in NF2-related schwannomatosis vestibular schwannoma
Source: Nat Commun. 2025 Mar 26;16:2944. doi: 10.1038/s41467-025-57586-z (PMC11947219; doi:10.1038/s41467-025-57586-z)
Supplement: Supplementary file 1 — Supplementary Information [file 41467_2025_57586_MOESM1_ESM.pdf]

Supplementary Table 1: Antibody clones and sources

Acronym	Clone	Source	Identifier
Recombinant Anti-S100 beta antibody (BSA and Azide free)	EP1576Y	Abcam	ab215989
Purified anti-Pan-Cytokeratin Antibody	AE-1/AE-3	BioLegend	914204
CD45 Monoclonal Antibody	CD45-2B11	eBioscience	14-9457-82
Recombinant Anti-ICAM1 antibody (BSA and Azide free)	EP1442Y	Abcam	ab271852
Recombinant Anti-Granzyme B antibody (BSA and Azide free)	EPR20129-217	Abcam	ab219803
Recombinant Anti-CD16 antibody (BSA and Azide free)	SP175	Abcam	ab243925
Anti-CX3CR1 antibody	Polyclonal	Abcam	ab8020
Recombinant Anti-CD11b antibody (BSA and Azide free)	EP1345Y	Abcam	ab187537
Recombinant Anti-CD11c antibody (BSA and Azide free)	EP1347Y	Abcam	ab216655
CD14 Rabbit mAb	D7A2T	Cell Signalling Technology	56082BF
Anti Iba1, Rabbit (for Immunocytochemistry)	Polyclonal	FUJIFILM Wako Pure Chemical Corp.	019-19741
Purified anti-human CD74 Antibody	LN2	BioLegend	326802
Anti-HLA-DR antibody	TAL 1B5	Abcam	ab20181
CD206/MRC1 Rabbit mAb	E2L9N	Cell Signalling Technology	91992
Purified anti-CD68 Antibody	KP1	BioLegend	916104
Rat anti-Human CD3	CD3-12	Bio-Rad	MCA1477
Recombinant Anti-CD4 antibody (BSA and Azide free)	EPR6855	Abcam	ab181724
CD8α Monoclonal Antibody	C8/144B	eBioscience	14-0085-82
Purified anti-human CD45RA Antibody	HI100	BioLegend	304102
Purified anti-human CD45RO Antibody	UCHL1	BioLegend	304202
Recombinant Anti-FOXP3 antibody (BSA and Azide free)	236A/E7	Abcam	ab96048
Mouse anti-Human Actin Alpha (Smooth Muscle)	1A4	Bio-Rad	MCA5781GA
CD31/PECAM-1 Antibody - BSA Free	JC/70A	Novus Biologicals	NB600-562
Anti-Human Von Willebrand Factor	Polyclonal	DAKO, Agilent	A0082
Purified anti-human CD235ab Antibody	HIR2	BioLegend	306602
Anti-HLA Class 1 ABC antibody	EMR8-5	Abcam	ab70328
Vimentin Antibody - BSA Free	RV202	Novus Biologicals	NBP1-97672
Recombinant Anti-HIF-1 alpha antibody (BSA and Azide free)	EP1215Y	Abcam	ab210073
VENTANA PD-L1 Rabbit Monoclonal Primary Antibody	SP263	Roche Diagnostics	07494190001
Recombinant Anti-Ki67 antibody (BSA and Azide free)	B56	Abcam	ab279657
Phospho-p44/42 MAPK (Erk1/2) (Thr202/Tyr204) XP® Rabbit mAb	D13.14.4E	Cell Signalling Technology	4370
MCT4 Polyclonal antibody	Polyclonal	Proteintech	#22787-1-AP
Purified anti-mouse/human CD44 Antibody	IM7	BioLegend	103001
Goat anti-Rabbit IgG (H+L) Cross-Adsorbed Secondary Antibody, Alexa Fluor™ 488	Polyclonal	ThermoFisher	A-11008

Supplementary Figure 1

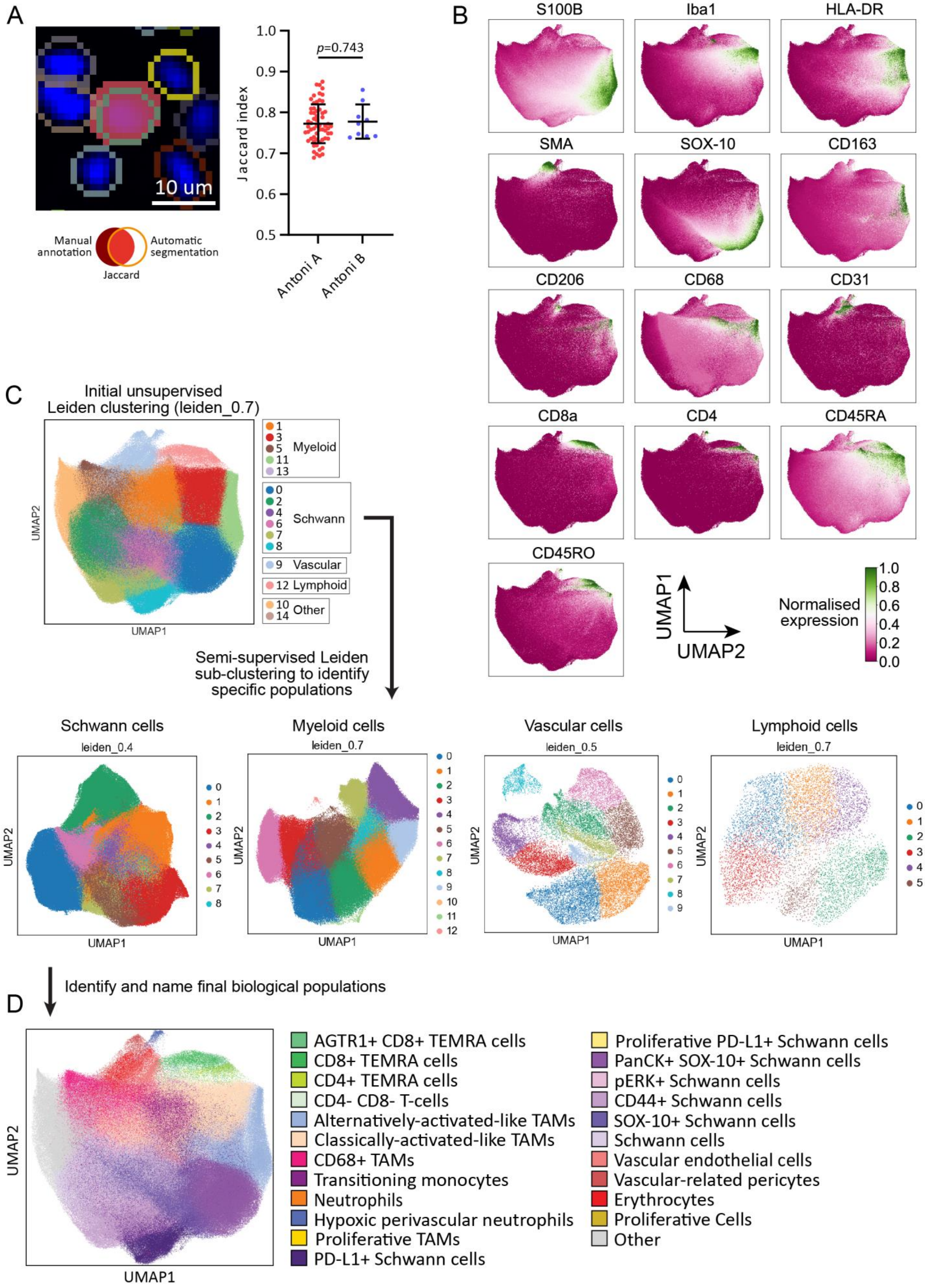

Supplementary Figure 1: Imaging mass cytometry marker visualisation and population clustering.

A: Jaccard analysis (which compares the manual vs automatic segmentation of the same cells), demonstrating accurate segmentation, and similar performance of our segmentation between histological regions (n=13). **B:** UMAPs of representative markers for core microenvironmental populations across all cells. Markers are normalised to the 99.9th percentile of their expression. Diverging scale indicating normalised expression (N.E.). **C:** Schematic of Leiden clustering methods for cell population derivation for IMC data. Parental population clusters from the original UMAP (for Schwann, myeloid, vascular and lymphoid populations) underwent a second round of focussed individual sub-clustering. After this second round of sub-clustering, clusters were given biological identities, with some clusters subsequently merged if they were biologically equivalent. **D:** UMAP of all identified populations generated from Leiden clustering methodology. Source data are provided as a Source Data file.

Supplementary Figure 2

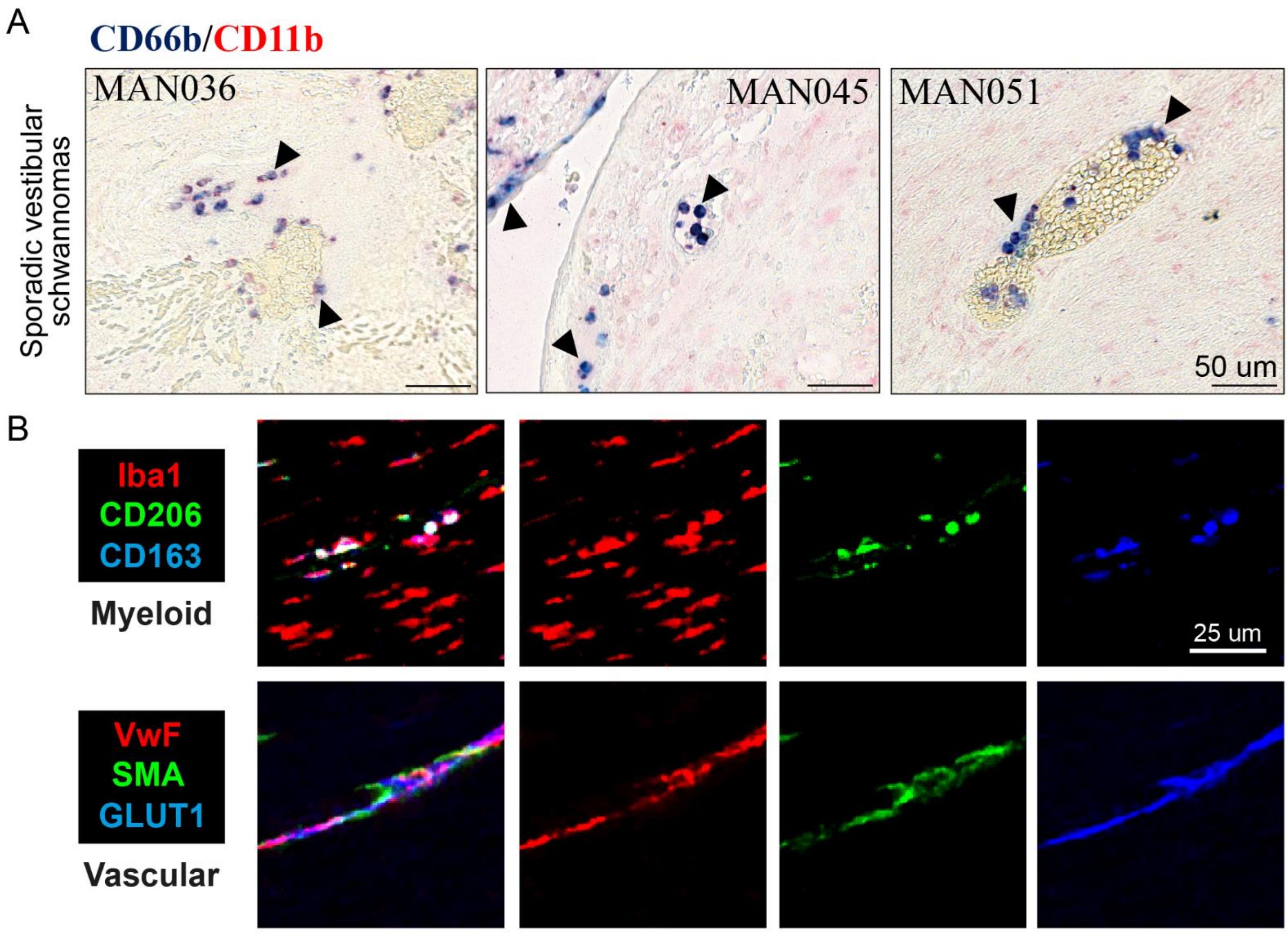

Supplementary Figure 2: Identification of vascular-associated CD66b^{+} neutrophils and CD206^{+} myeloid cell populations within VS tumours

A: Representative immunohistochemistry images showing identification of CD66b^{+}CD11b^{hi} cells (neutrophils) within sporadic VS tumours (n=3). Neutrophils appear mainly vascular associated. **B:** Representative IMC images of NF2 SWN-VS tumours showing expression of (top) CD206 in combination with alternatively activated-like macrophage markers Iba1 and CD163 and (bottom) of vascular associated markers VwF, SMA and GLUT1. CD206^{+} myeloid cells are exclusively vascular-associated.

Supplementary Figure 3

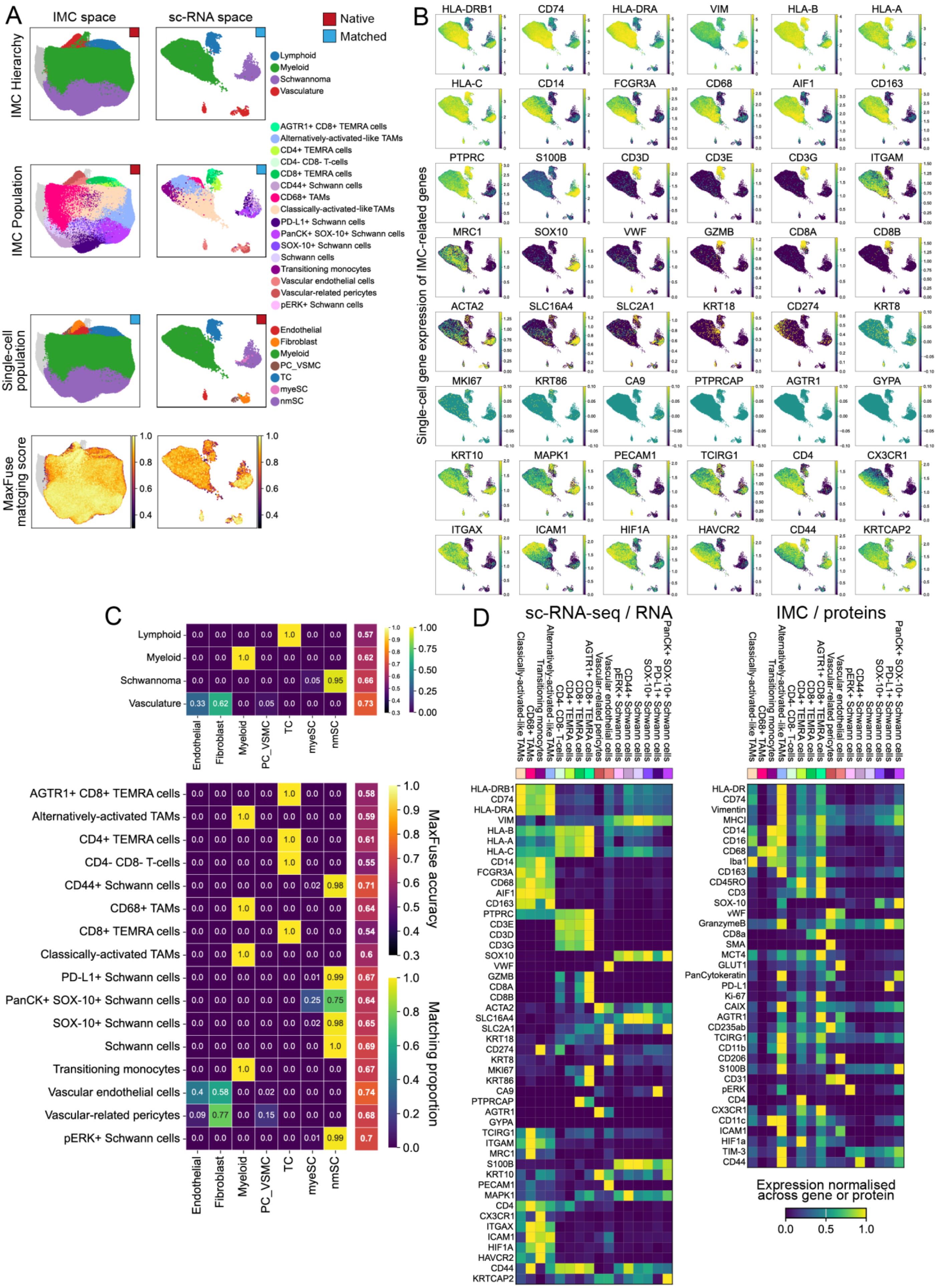

	Endothelial	Fibroblast	Myeloid	PC_VSMC	TC	myeSC	nmSC	MaxFuse accuracy
Lymphoid	0.0	0.0	0.0	0.0	1.0	0.0	0.0	0.57
Myeloid	0.0	0.0	1.0	0.0	0.0	0.0	0.0	0.62
Schwannoma	0.0	0.0	0.0	0.0	0.0	0.05	0.95	0.66
Vasculature	0.33	0.62	0.0	0.05	0.0	0.0	0.0	0.73

	Endothelial	Fibroblast	Myeloid	PC_VSMC	TC	myeSC	nmSC	MaxFuse accuracy
AGTR1+ CD8+ TEMRA cells	0.0	0.0	0.0	0.0	1.0	0.0	0.0	0.58
Alternatively-activated TAMs	0.0	0.0	1.0	0.0	0.0	0.0	0.0	0.59
CD4+ TEMRA cells	0.0	0.0	0.0	0.0	1.0	0.0	0.0	0.61
CD4- CD8- T-cells	0.0	0.0	0.0	0.0	1.0	0.0	0.0	0.55
CD44+ Schwann cells	0.0	0.0	0.0	0.0	0.0	0.02	0.98	0.71
CD68+ TAMs	0.0	0.0	1.0	0.0	0.0	0.0	0.0	0.64
CD8+ TEMRA cells	0.0	0.0	0.0	0.0	1.0	0.0	0.0	0.54
Classically-activated TAMs	0.0	0.0	1.0	0.0	0.0	0.0	0.0	0.6
PD-L1+ Schwann cells	0.0	0.0	0.0	0.0	0.0	0.01	0.99	0.67
PanCK+ SOX-10+ Schwann cells	0.0	0.0	0.0	0.0	0.0	0.25	0.75	0.64
SOX-10+ Schwann cells	0.0	0.0	0.0	0.0	0.0	0.02	0.98	0.65
Schwann cells	0.0	0.0	0.0	0.0	0.0	0.0	1.0	0.69
Transitioning monocytes	0.0	0.0	1.0	0.0	0.0	0.0	0.0	0.67
Vascular endothelial cells	0.4	0.58	0.0	0.02	0.0	0.0	0.0	0.74
Vascular-related pericytes	0.09	0.77	0.0	0.15	0.0	0.0	0.0	0.68
pERK+ Schwann cells	0.0	0.0	0.0	0.0	0.0	0.01	0.99	0.7

Supplementary Figure 3: Single-cell alignment of IMC and scRNA-seq datasets for population cluster validition.

MaxFuse was used to pair cells between IMC (n=13) and scRNA-seq (n=11) modalities using shared features (proteins and genes). **A**: Once cells were matched, labels could be transferred between modalities, allowing the distribution of populations to be compared in IMC and scRNA-seq space, and showing a concordance between the parental cell populations between modalities. Native (red box) indicates the modality in which the labels were originally assigned, and transferred (blue box) indicates how population labels were transferred from the native modality (from individually matched cells) onto the cells in the secondary modality. MaxFuse gives a score for the accuracy of matching, with 0.3 set at the minimum required for matching. Matching of some cells was not attempted (in grey, 9.56% of IMC cells), because the cells did not have a clear phenotype ('Other' in IMC), or because they were not present in the scRNA-seq dataset (e.g. neutrophils). **B:** UMAP projections of the expression of the genes for the proteins in the IMC panel in the scRNA-seq dataset. **C:** Confusion matrix comparing the cell population labels assigned in the two modalities, using either parental or more granular labels from IMC (vertical) compared with scRNA-seq populations defined in source manuscript[30] (horizontal). Mean MaxFuse accuracy scores were calculated within each population, showing accurate mapping across population. **D:** Heatmap of the IMC markers in the IMC population, alongside the genes for those proteins in the matched scRNA-seq cells. Protein and gene heatmaps have different number of rows because some markers detected using a single antibody (e.g. pan-cytokeratin) are transcribed by several genes. Source data are provided as a Source Data file.

Supplementary Figure 4

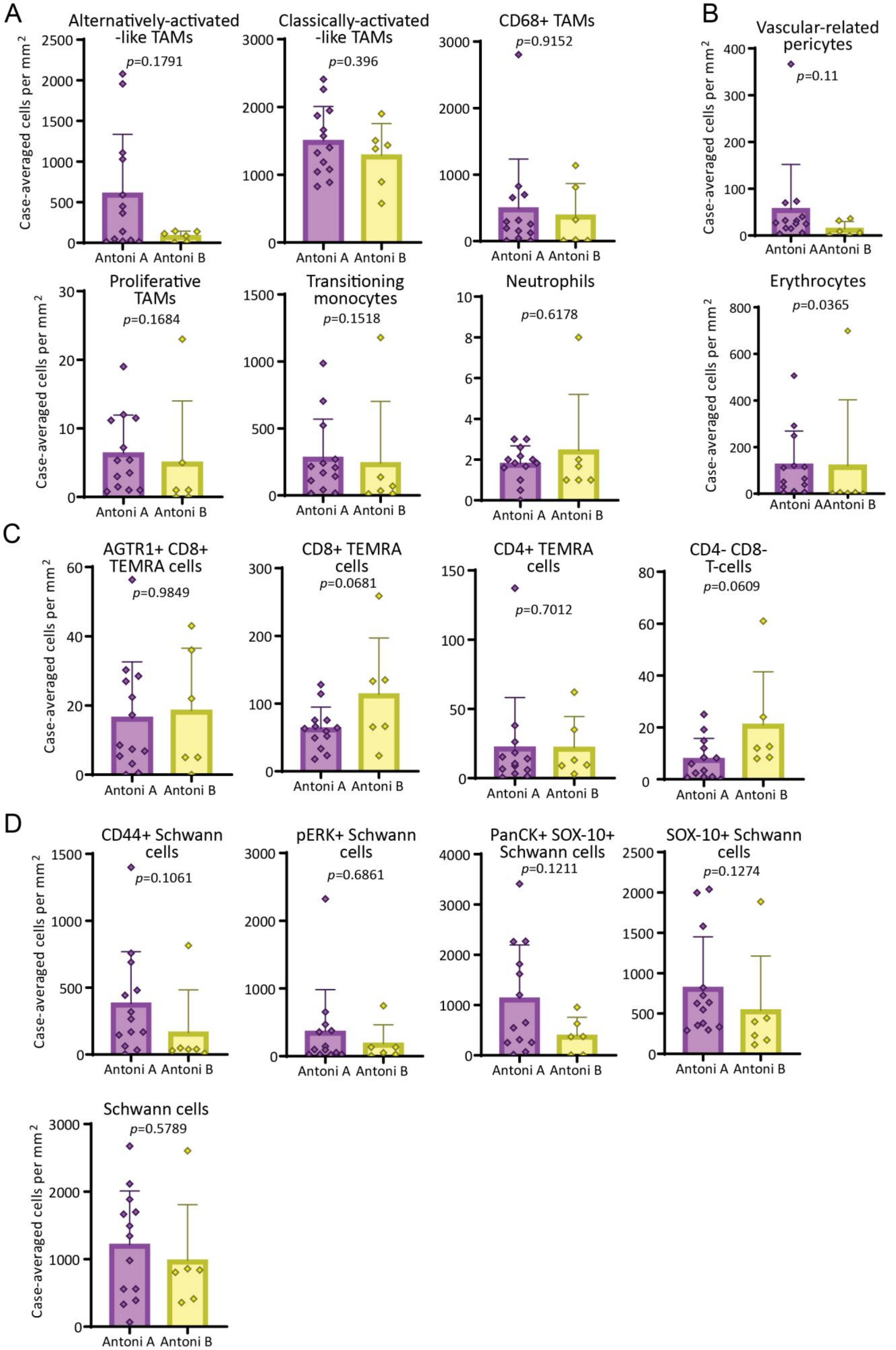

Supplementary Figure 4: Comparison of case-averaged abundance of cell populations across Antoni A and Antoni B regions.

A: Comparison of myeloid cell populations. **B:** Comparison of vascular-related populations. **C:** Comparison of T-cell populations**. D:** Comparison of Schwann cell populations. (**A-D**) Statistical comparisons were made using two-tailed unpaired t-tests (for normally distributed data) or two-tailed Mann Whitney U tests (n=13 cases analysed; 12 cases represented for Antoni A and 6 different cases represented for Antoni B). Results are the mean + S.D. Source data are provided as a Source Data file.

Supplementary Figure 5

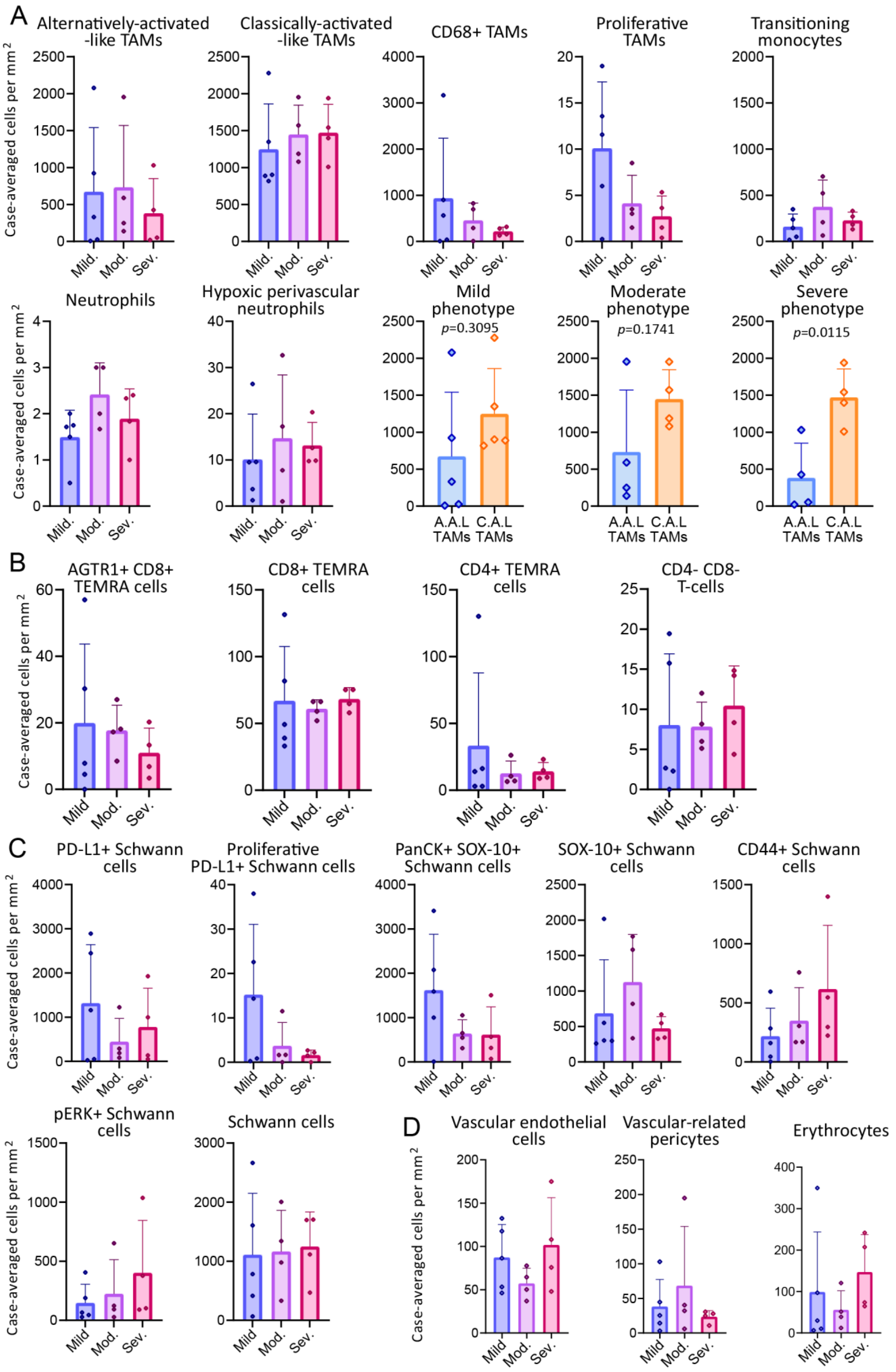

Supplementary Figure 5: Comparison of case-averaged abundance of cell populations between genetic disease severity groups.

A: Comparison of myeloid cell populations. **B:** Comparison of T-cell populations. **C:** Comparison of Schwann cell populations**.** **D:** Comparison of vascular-related cell populations. Statistical comparisons were made for two group comparisons using unpaired two-tailed t-tests (for normally distributed data) or two-tailed Mann Whitney U tests. Statistical comparisons were made for three group comparisons using one way Anova with Tukey's post-hoc test (for normally distributed data) or Kruskall Wallis with Dunn's post-hoc Test. Total n=13 for sample set; n= 5 for mild disease, n = 4 for moderate disease and n=4 for severe disease as specified in Table 1. Results are the mean + S.D. Source data are provided as a Source Data file.

Supplementary Figure 6

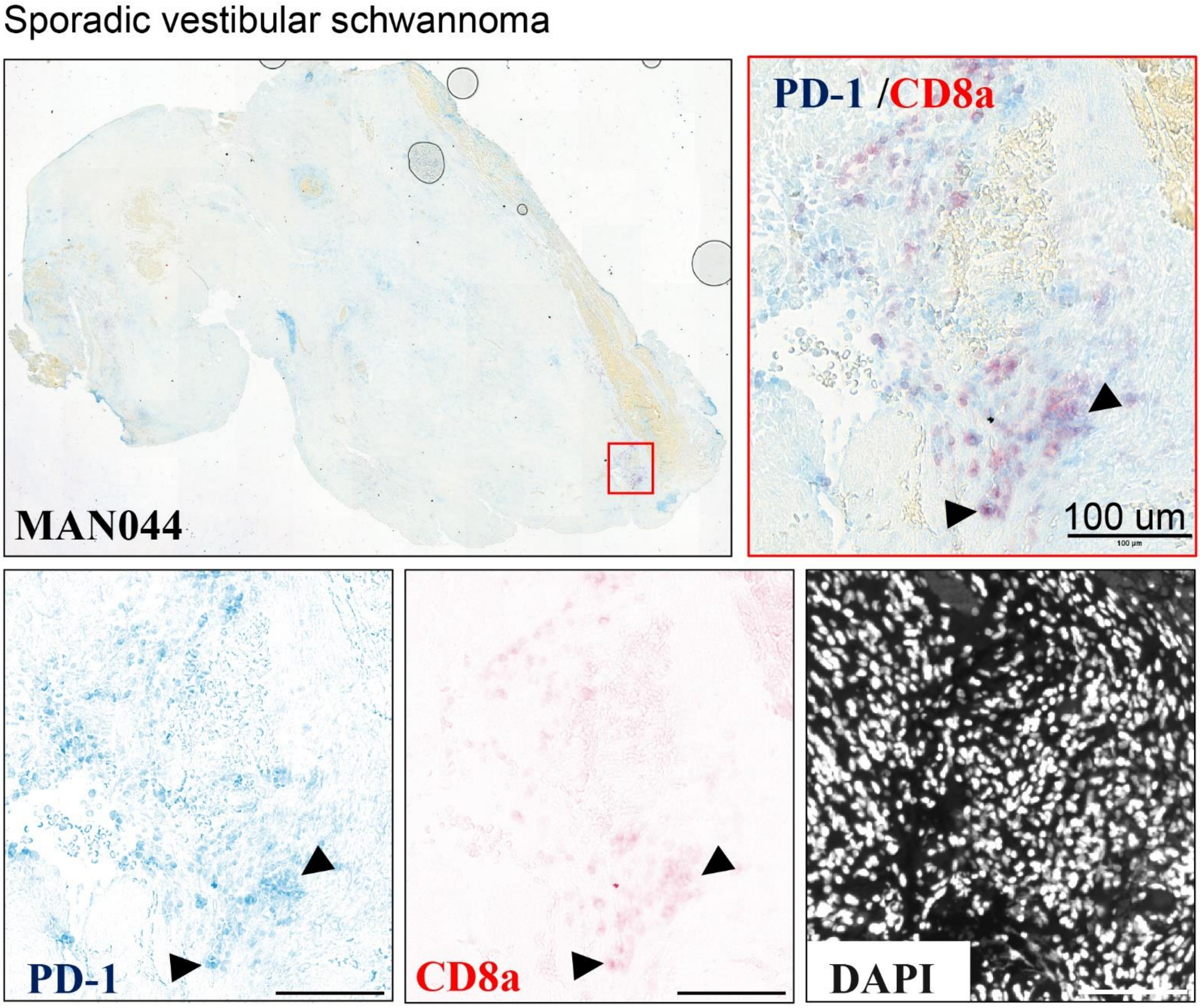

Supplementary Figure 6: Identification of PD-1^{+} CD8^{+} T cells within VS tumours

Representative immunohistochemistry images showing identification of PD-1^{+}CD8^{+} cells within a sporadic VS tumours (showing one of three analysed cases). Arrow heads identify PD-1^{+}CD8^{+} T cells.

Supplementary Figure 7

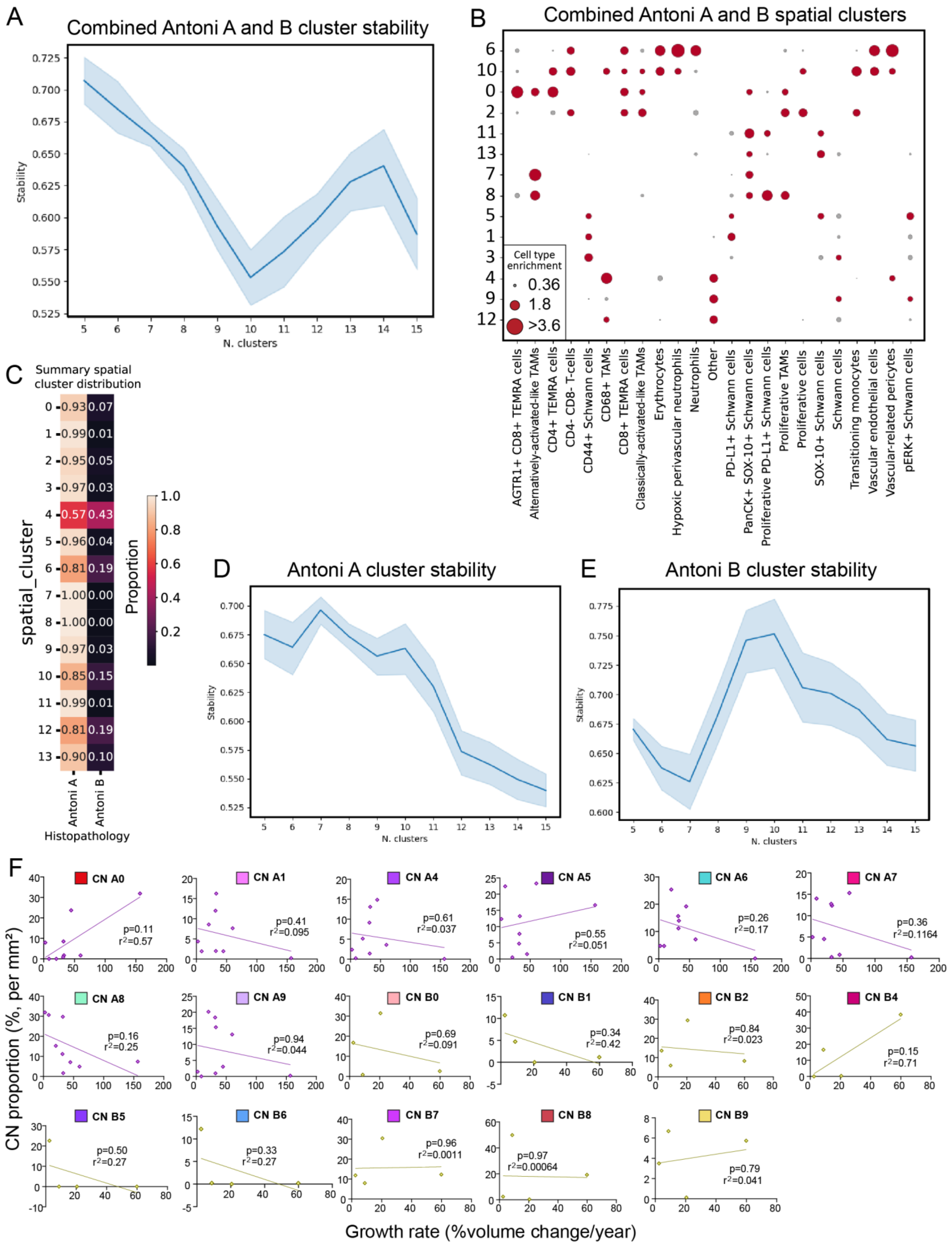

Supplementary Figure 7: Generation of cellular neighbourhoods (CNs)

A: Elbow plot inferring cluster stability of combined Antoni A and B regions of interest (ROIs). **B:** Dot plot of spatial clusters from combined Antoni A and B ROIs. **C:** Heatmap of spatial cluster distribution across Antoni A and B ROIs, indicating the loss of Antoni B spatial signatures. **D:** Elbow plot inferring cluster stability of Antoni A-only ROIs**. E:** Elbow plot inferring cluster stability of Antoni B-only ROIs. **F:** Correlations between CNs and tumour growth rate. Correlations were assessed by either Pearson correlation coefficient or Spearman correlation. Line of best-fit was generated by simple linear regression analysis. Source data are provided as a Source Data file.

Supplementary Figure 8

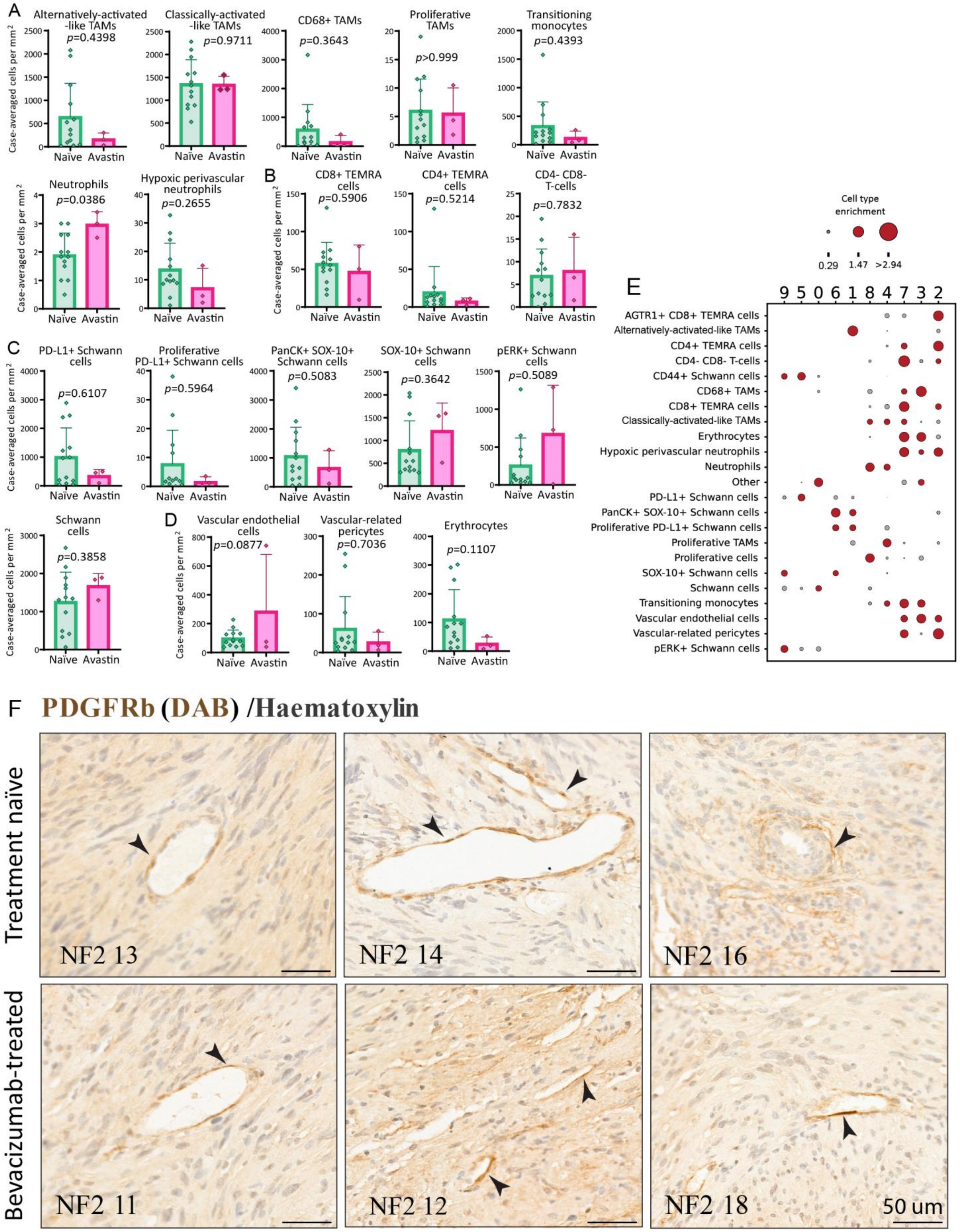

Supplementary Figure 8: Effects of bevacizumab-treatment on the microenvironment of vestibular schwannoma.

A: Comparison of myeloid cell populations. **B:** Comparison of T-cell populations. **C:** Comparison of Schwann cell populations**.** **D:** Comparison of vascular-related cell populations. **E:** Dot plot of spatial clusters from neighbourhood

analyses using CellCharter in combined treatment naïve and bevacizumab-treated cases. Statistical comparisons were made using two two-tailed unpaired t-tests (for normally distributed data) or two-tailed Mann Whitney U tests. **F**: Representative immunohistochemistry images showing identification of PDGFRβ$^+$ cells (pericytes) associated with the vasculature within cases of treatment naïve and bevacizumab-treated *NF2* SWN VS tumours. Arrow heads identify PDGFRβ$^+$ cells (n=3 per group). **(A-D)** Results are the mean + S.D (n=13 for treatment naïve and n=3 for bevacizumab-treated groups). Source data are provided as a Source Data file.
