# Supplementary material for: Spatial mapping of immune cell environments in NF2-related schwannomatosis vestibular schwannoma
Source: Nat Commun. 2025 Mar 26;16:2944. doi: 10.1038/s41467-025-57586-z (PMC11947219; doi:10.1038/s41467-025-57586-z)
Supplement: Supplementary file 2 — Reporting Summary [file 41467_2025_57586_MOESM2_ESM.pdf]

nature portfolio

Corresponding author(s): Kevin Couper

Last updated by author(s): Feb 2, 2025

Reporting Summary

Nature Portfolio wishes to improve the reproducibility of the work that we publish. This form provides structure for consistency and transparency in reporting. For further information on Nature Portfolio policies, see our Editorial Policies and the Editorial Policy Checklist.

Statistics

For all statistical analyses, confirm that the following items are present in the figure legend, table legend, main text, or Methods section.

n/a	Confirmed	
☐	☒	The exact sample size (*n*) for each experimental group/condition, given as a discrete number and unit of measurement
☐	☒	A statement on whether measurements were taken from distinct samples or whether the same sample was measured repeatedly
☐	☒	The statistical test(s) used AND whether they are one- or two-sided<br>*Only common tests should be described solely by name; describe more complex techniques in the Methods section.*
☐	☒	A description of all covariates tested
☐	☒	A description of any assumptions or corrections, such as tests of normality and adjustment for multiple comparisons
☐	☒	A full description of the statistical parameters including central tendency (e.g. means) or other basic estimates (e.g. regression coefficient) AND variation (e.g. standard deviation) or associated estimates of uncertainty (e.g. confidence intervals)
☒	☐	For null hypothesis testing, the test statistic (e.g. *F*, *t*, *r*) with confidence intervals, effect sizes, degrees of freedom and *P* value noted<br>*Give P values as exact values whenever suitable.*
☒	☐	For Bayesian analysis, information on the choice of priors and Markov chain Monte Carlo settings
☒	☐	For hierarchical and complex designs, identification of the appropriate level for tests and full reporting of outcomes
☐	☒	Estimates of effect sizes (e.g. Cohen's *d*, Pearson's *r*), indicating how they were calculated

Our web collection on statistics for biologists contains articles on many of the points above.

Software and code

Policy information about availability of computer code

Data collection	N/A
Data analysis	-IMC denoise for raw image enhancement (open source), v1.0.0, https://github.com/PENGLU-WashU/IMC_Denoise/<br>-IMC segmentation pipeline for single-cell data extraction from raw IMC images (open source), v3.5, https://github.com/BodenmillerGroup/ImcSegmentationPipeline<br>-IMC data analysis pipeline for processing, quality control, cell clustering and phenotyping, and single-cell and spatial analyses, v0.99.0, https://github.com/BodenmillerGroup/IMCDataAnalysis<br>-SpOOx spatial omics pipeline for cell-cell interaction spatial analyses, first release version, https://github.com/Taylor-CCB-Group/SpOOx<br>-CellCharter package for spatial cluster identification and cellular neighbourhood derivation, v0.2.0, https://github.com/CSOgroup/cellcharter<br>- Maxfuse package (https://github.com/shuxiaoc/maxfuse/tree/main/maxfuse) for IMC and scRNA-seq data integration<br>- Receptor ligand analysis with performed using Squidpy (https://github.com/scverse/squidpy)

For manuscripts utilizing custom algorithms or software that are central to the research but not yet described in published literature, software must be made available to editors and reviewers. We strongly encourage code deposition in a community repository (e.g. GitHub). See the Nature Portfolio guidelines for submitting code & software for further information.

Data

Policy information about availability of data

All manuscripts must include a data availability statement. This statement should provide the following information, where applicable:

- Accession codes, unique identifiers, or web links for publicly available datasets
- A description of any restrictions on data availability
- For clinical datasets or third party data, please ensure that the statement adheres to our policy

All data supporting the findings of this study are available within the paper and its Supplementary Information. All raw data has been uploaded to DataDryad with links provided. Clinical information regarding participants is provided in Table 1, and antibody clones and sources are provided in Supplementary Table 1. All methodologies used for this study are publicly available on GitHub and are cited within this paper.

Research involving human participants, their data, or biological material

Policy information about studies with human participants or human data. See also policy information about sex, gender (identity/presentation), and sexual orientation and race, ethnicity and racism.

Reporting on sex and gender	Our study had equivalent numbers of both sexes (8 male and 8 female). We considered the inclusion of both sexes and that findings apply to both sexes, given that existing literature (e.g. PMID: 32470937) does not suggest there are significant differences in the pathogenesis of NF2 SWN-related VS that is sex-driven.
Reporting on race, ethnicity, or other socially relevant groupings	N/A
Population characteristics	Age range at diagnosis (12 years - 57 years) and surgery (17 years - 59 years).<br>NF2 SWN disease severity states are based upon presence of NF2 pathogenic variants: 5 mild, 5 moderate, 6 severe phenotypes.<br>3 patients previously had bevacizumab treatment, and 2 patients had prior resections.<br>4 sporadic vestibular schwannoma cases were used solely for immunohistochemistry.
Recruitment	Retrospective samples were used in this study with no recruitment of new patients with access to retrospective samples in line with Medical Research Council, UK, guidelines. Vestibular schwanomma patients were initially selected for sample banking on the basis of a clinical NF2 Schwannomatosis (NF2 SWN) diagnosis, and where tumour progression / symptoms necessitated surgery. There is a natural bias in the participant population towards patients with large, growing tumours. However, we believe this is justified as surgery is typically not performed on smaller, static tumours in NF2 SWN patients due to the considerable associated morbidity.
Ethics oversight	This study was approved by the HRA and HCRW. REC: 20/NW/0015. IRAS ID: 274046.

Note that full information on the approval of the study protocol must also be provided in the manuscript.

Field-specific reporting

Please select the one below that is the best fit for your research. If you are not sure, read the appropriate sections before making your selection.

[x] Life sciences [ ] Behavioural & social sciences [ ] Ecological, evolutionary & environmental sciences

For a reference copy of the document with all sections, see nature.com/documents/nr-reporting-summary-flat.pdf

Life sciences study design

All studies must disclose on these points even when the disclosure is negative.

Sample size	NF2 SWN is a rare disease and sample acquisition is limited and time-consuming (i.e. our sample set was over a retrospective period of 10+ years). Based on previous published studies (PMID: 32470937, PMID: 37680691), we anticipated a minimum of 10 samples would be appropriate for this study.
Data exclusions	Some participant samples were excluded due to previous stereotactic radiosurgery (1 case). Additionally, some regions of interest were excluded if staining was poor (~5 regions). FOXP3 and PD-1 staining were also excluded from analyses due to non-specific staining and failed staining, respectively.
Replication	Multiple regions of interest were sampled per case to account for intra-tumoral heterogeneity, as well as accumulative inter-tumoral heterogeneity.
Randomization	N/A
Blinding	N/A

Reporting for specific materials, systems and methods

We require information from authors about some types of materials, experimental systems and methods used in many studies. Here, indicate whether each material, system or method listed is relevant to your study. If you are not sure if a list item applies to your research, read the appropriate section before selecting a response.

Materials & experimental systems

n/a	Involved in the study
☐	☒ Antibodies
☒	☐ Eukaryotic cell lines
☒	☐ Palaeontology and archaeology
☒	☐ Animals and other organisms
☒	☐ Clinical data
☒	☐ Dual use research of concern
☒	☐ Plants

Methods

n/a	Involved in the study
☒	☐ ChIP-seq
☒	☐ Flow cytometry
☒	☐ MRI-based neuroimaging

Antibodies

Antibodies used

Antibodies used for IHC:

Name – clone – supplier – catalogue number
Recombinant anti-S100 beta antibody (BSA and azide free) – EP1576Y – Abcam – ab215989
Purified anti-Pan-Cytokeratin antibody – AE-1/AE-3 – Biolegend – 914204
CD45 monoclonal antibody – CD45-2B11 – eBioscience – 14-9457-82
Recombinant anti-ICAM1 antibody (BSA and azide free) – EP1442Y – Abcam – ab271852
Recombinant anti-Granzyme B antibody (BSA and azide free) – EPR20129-217 – Abcam – ab219803
Recombinant anti-CD16 antibody (BSA and azide free) – SP175 – Abcam – ab243925
anti-CX3CR1 antibody – polyclonal – Abcam – ab8020
Recombinant anti-CD11b antibody (BSA and azide free) – EP1345Y – Abcam – ab187537
Recombinant anti-CD11c antibody (BSA and azide free) – EP1347Y – Abcam – ab216655
CD14 rabbit mAb – D7A2T – Cell Signalling Technologies – 56082BF
Anti-Iba1, Rabbit – polyclonal – FUJIFILM Wako Pure Chemical Corp. – 019-19741
Purified anti-human CD74 antibody – LN2 – Biolegend – 326802
Anti-HLADR antibody – TAL-1B5 – Abcam – ab20181
CD206/MRC1 rabbit mAb – E2L9N - Cell Signalling Technologies – 91992
Purified anti-CD68 antibody – KP1 – Biolegend – 916104
Purified anti-human CD3 – CD3-12 – Bio-Rad – MCA1477
Recombinant anti-CD4 antibody (BSA and azide free) – EPR6855 – Abcam – ab181724
CD8α monoclonal antibody – C8/144B – eBioscience – 14-0085-82
Purified anti-human CD45RA antibody – H1100 – Biolegend – 304102
Purified anti-human CD45RO antibody – UCHL1 – Biolegend – 304202
Recombinant anti-FOXP3 antibody (BSA and azide free) – 236A/E7 – Abcam – ab96048
Mouse anti-Human Actin Alpha (Smooth Muscle) – 1A4 – Bio-Rad – MCA5781GA
CD31/PECAM-1 antibody (BSA free) – JC/70A – Novus Biologicals – NB600-562
Anti-Human Von Willebrand Factor – polyclonal – DAKO, Agilent – A0082
Purified anti-Human CD235ab antibody – H1R2 – Biolegend – 306602
Anti-HLA Class I ABC antibody – EMR8-5 – Abcam – ab70328
Vimentin antibody (BSA free) – RV202 – Novus Biologicals – NBP1-97672
Recombinant anti-HIF-1 alpha antibody (BSA and azide free) – EP1215Y – Abcam – ab210073
VENTANA PD-L1 rabbit monoclonal primary antibody – SP263 – Roche Diagnostics – 07494190001
Recombinant anti-Ki-67 antibody (BSA and azide free) – B56 – Abcam – ab279657
Phospho-p44/42 MAPK (Erk1/2) (Thr202/Tyr204) XP Rabbit mAb – D13.14.4E – Cell Signalling Technologies – 4370
MCT4 antibody – polyclonal – Proteintech - #22787-1-AP
Purified anti-mouse/human CD44 antibody – 1M7 – Biolegend – 103001
Goat anti-rabbit IgG (H+L) cross-absorbed secondary antibody, Alexa Fluor 488 – polyclonal -ThermoFisher – A-11008

Antibodies using for immunohistochemistry:
PDGFRb, abcam, ab69506, clone# 42G12
PD-1 abcam ab137132 clone# EPR4877(2)
CD8a Biolegend 372902 clone# c8/144B
CD66b BD Pharmingen 555723 clone# G10F5
CD11b abcam ab187537 clone# EP1345Y

Validation	Antibodies were validated using optimisation tissues including human spleen, lymph node, brain and tumour biopsies.

Plants

Seed stocks	N/A
Novel plant genotypes	N/A
Authentication	N/A
